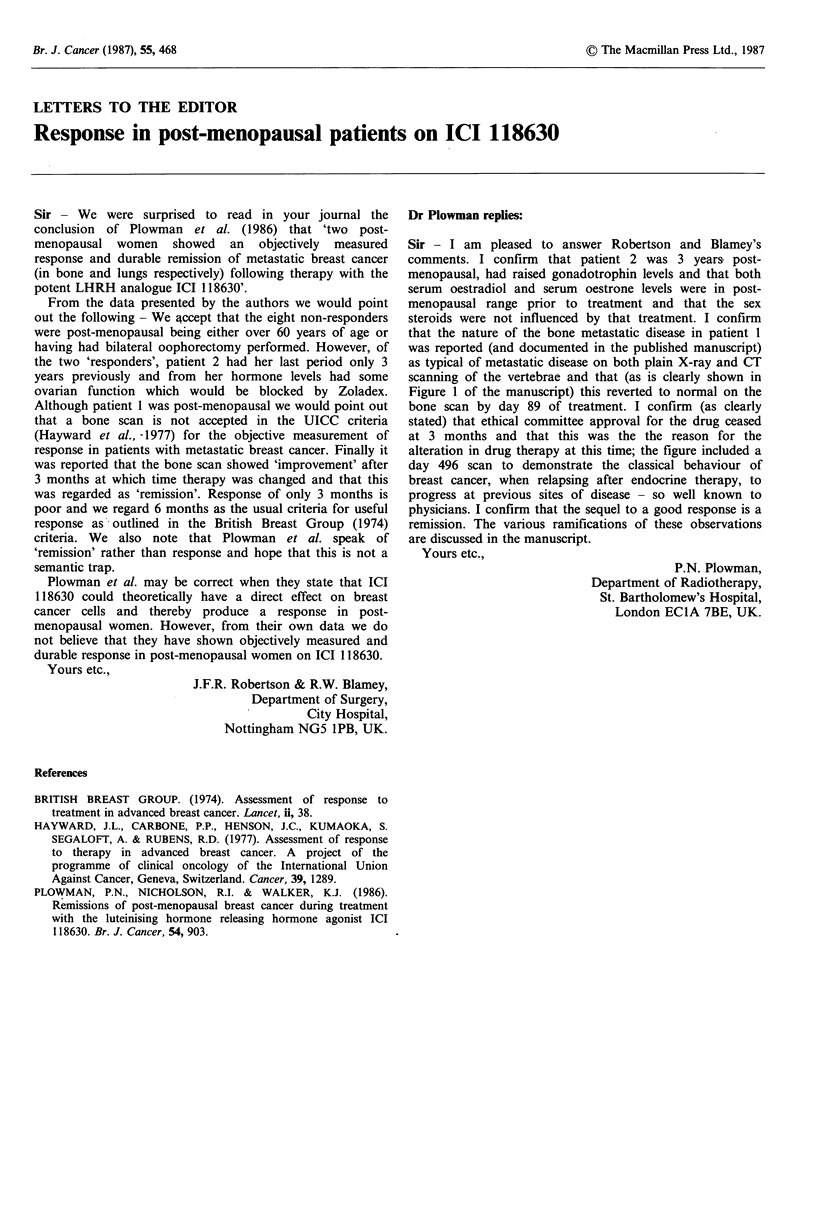# Dr Plowman replies

**Published:** 1987-04

**Authors:** P.M. Plowman


**Dr Plowman replies:**

**Sir** – I am pleased to answer Robertson and Blamey's comments. I confirm that patient 2 was 3 years post-menopausal, had raised gonadotrophin levels and that both serum oestradiol and serum oestrone levels were in post-menopausal range prior to treatment and that the sex steroids were not influenced by that treatment. I confirm that the nature of the bone metastatic disease in patient 1 was reported (and documented in the published manuscript) as typical of metastatic disease on both plain X-ray and CT scanning of the vertebrae and that (as is clearly shown in Figure 1 of the manuscript) this reverted to normal on the bone scan by day 89 of treatment. I confirm (as clearly stated) that ethical committee approval for the drug ceased at 3 months and that this was the the reason for the alteration in drug therapy at this time; the figure included a day 496 scan to demonstrate the classical behaviour of breast cancer, when relapsing after endocrine therapy, to progress at previous sites of disease – so well known to physicians. I confirm that the sequel to a good response is a remission. The various ramifications of these observations are discussed in the manuscript.

Yours etc.,

P.N. Plowman,
Department of Radiotherapy,
St. Bartholomew's Hospital,
London EC1A 7BE, UK.